# Risk profiles and pattern of antithrombotic use in patients with non-valvular atrial fibrillation in Thailand: a multicenter study

**DOI:** 10.1186/s12872-018-0911-4

**Published:** 2018-08-25

**Authors:** Rungroj Krittayaphong, Arjbordin Winijkul, Komsing Methavigul, Wattana Wongtheptien, Chaiyasith Wongvipaporn, Treechada Wisaratapong, Rapeephon Kunjara-Na-Ayudhya, Smonporn Boonyaratvej, Chulalak Komoltri, Pontawee Kaewcomdee, Ahthit Yindeengam, Piyamitr Sritara, for the COOL-AF Investigators, Tomorn Thongsri, Kriengkrai Hengrussamee, Pattraporn Srirattana, Wattana Wongtheptien, Pornchai Ngamjanyaporn, Arintaya Phrommintikul, Smonporn Boonyaratavej, Pongpun Jittham, Treechada Wisaratapong, Sirin Apiyasawat, Arjbordin Winijkul, Rungroj Krittayaphong, Roj Rojjarekampai, Kulyot Jongpiputvanich, Somchai Dutsadeevettakul, Chaiyasith Wongvipaporn, Thanita Boonyapiphat, Weerapan Wiwatworapan, Khanchai Siriwattana, Eakarnantha Arnanththanitha, Watchara Konkaew, Thoranis Chantrarat, Kasem Ratanasumawong, Wiwat Kanjanarutjawiwat, Sakaorat Kornbongkotmas, Thanasak Patmuk, Praprut Thanakitcharu, Suchart Arunsiriwattana, Thaworn Choochunklin, Sumon Tangsuntornwiwat

**Affiliations:** 1 Division of Cardiology, Department of Medicine, Faculty of Medicine Siriraj Hospital, Mahidol University, 2 Wanglang Road, Bangkoknoi, Bangkok, 10700 Thailand; 2 Department of Cardiology, Central Chest Institute of Thailand, Nonthaburi, Thailand; 3 Chiangrai Prachanukroh Hospital, Chiang Rai, Thailand; 4 Srinakarind Hospital, Faculty of Medicine, Khon Kaen University, Khon Kaen, Thailand; 5 Faculty of Medicine, Prince of Songkla University, Songkla, Thailand; 6 Vichaiyut Hospital and Medical Center, Bangkok, Thailand; 7 Faculty of Medicine, Chulalongkorn University, Bangkok, Thailand; 8 Department of Research Promotion, Faculty of Medicine Siriraj Hospital, Mahidol University, Bangkok, Thailand; 9 Faculty of Medicine Ramathibodi Hospital, Mahidol University, Bangkok, Thailand

**Keywords:** Risk profiles, Antithrombotics, Non-valvular atrial fibrillation, Thailand

## Abstract

**Background:**

Anticoagulation therapy is a standard treatment for stroke prevention in patients with non-valvular atrial fibrillation (NVAF) that have risk factors for stroke. However, anticoagulant increases the risk of bleeding, especially in Asians. We aimed to investigate the risk profiles and pattern of antithrombotic use in patients with NVAF in Thailand, and to study the reasons for not using warfarin in this patient population.

**Methods:**

A nationwide multicenter registry of patients with NVAF was created that included data from 24 hospitals located across Thailand. Demographic data, atrial fibrillation-related data, comorbid conditions, use of antithrombotic drugs, and reasons for not using warfarin were collected. Data were recorded in a case record form and then transferred into a web-based system.

**Results:**

A total of 3218 patients were included. Average age was 67.3 ± 11.3 years, and 58.2% were male. Average CHADS_2_, CHA_2_DS_2_-VASc, and HAS-BLED score was 1.8 ± 1.3, 3.0 ± 1.7, and 1.5 ± 1.0, respectively. Antiplatelet was used in 26.5% of patients, whereas anticoagulant was used in 75.3%. The main reasons for not using warfarin in those with CHA_2_DS_2_-VASc ≥2 included already taking antiplatelet (26.6%), patient preference (23.1%), and using non-vitamin K antagonist oral anticoagulants (NOACs) (22.7%). Anticoagulant was used in 32.3% of CHA_2_DS_2_-VASc 0, 56.8% of CHA_2_DS_2_-VASc 1, and 81.6% of CHA_2_DS_2_-VASc ≥2. The use of NOACs increased from 1.9% in 2014 to 25.6% in 2017.

**Conclusions:**

Anticoagulation therapy was prescribed in 75.3% of patients with NVAF. Among those receiving anticoagulant, 90.9% used warfarin and 9.1% used NOACs. The use of NOACs increased over time.

**Electronic supplementary material:**

The online version of this article (10.1186/s12872-018-0911-4) contains supplementary material, which is available to authorized users.

## Background

Non-valvular atrial fibrillation (NVAF) is a common cardiac arrhythmia in clinical practice with a prevalence of approximately 1–2% [1, 2] which may be higher in patients with structural disease [3]. NVAF create a slow-flow situation within the atrium especially left atrial appendage leading to thrombus formation and thromboembolic event [1]. Current practice guidelines recommend the use of anticoagulant in patients with NVAF that have additional risk factor(s) for stroke [4, 5]. CHA_2_DS_2_VASc score has been recommended as a risk stratification tool for predicting stroke in this group [4]. The annual risk of ischemic stroke in patients with non-valvular atrial fibrillation (NVAF) may be higher than 5% in patients with a high CHA_2_DS_2_VASc score [1]. Warfarin is associated with many types of food- and drug-related interactions, so international normalized ratio (INR) monitoring is needed [6, 7]. Although there are many non-vitamin K antagonist oral anticoagulants (NOAC), such as direct thrombin inhibitor and factor Xa inhibitors, warfarin is still widely used in Asian, and in low and middle income countries [8, 9]. Although anticoagulation therapy can reduce ischemic stroke, it can also cause or contribute to major bleeding or intracerebral hemorrhage. Asian population was reported to have a higher risk of intracerebral hemorrhage, as a proportion of subtype of stroke, compared to Western population [2, 10]. Asian population also demonstrated a higher risk of warfarin-related intracerebral hemorrhage and bleeding-related complications [11, 12]. For a variety of reasons, anticoagulant is prescribed in less than half of patients with AF, including those in the intermediate- and high-risk groups [13, 14]. It is, therefore, important to study and understand the pattern of use of antithrombotic medication via the analysis of real-world data in this era.

Accordingly, the aim of this study was to investigate the risk profiles and pattern of antithrombotic use in patients with NVAF in Thailand, and to study the reasons for not using warfarin in this patient population.

## Methods

### Study population and data

NVAF patients were consecutively enrolled from 24 hospitals located all across Thailand. Thirteen of those centers are university hospitals, and ten are regional or general hospitals. The protocol for this study was approved by the institutional review boards (IRBs) of the Thailand Ministry of Public Health and IRB of each participating hospital namely Buddhachinaraj Hospital, Central Chest Institute of Thailand, Charoen Krung Pracha Rak Hospital, Chiangrai Prachanukroh Hospital, Chonburi Hospital, Chiang Mai Hospital, King Chulalongkorn Memorial Hospital, Naresuan University Hospital, Songklanakarind Hospital, Ramathibodi Hospital, Siriraj Hospital, Thammasat Hospital, Golden Jubilee Medical Center, Srinakarind Hospital, Lampang Hospital, Maharat Nakorn Ratchasima Hospital, Nakornping Hospital, Phramongkutklao Hospital, Police General Hospital, Prapokklao Hospital (Chanthaburi), Ratchaburi Hospital, Surat Thani Hospital, Surin Hospital, and Udonthani Hospital. All patients provided written informed consent prior to participation in this study. Patients aged ≥18 years with atrial fibrillation diagnosed by standard ECG or ambulatory monitoring were eligible for inclusion. Patients having one or more of the following were excluded: 1) ischemic stroke within 3 months; 2) thrombocytopenia (< 100,000/mm3), myeloproliferative disorders, hyperviscosity syndrome, or antiphospholipid syndrome; 3) prosthetic valve or valve repair; 4) rheumatic valve disease or significant valve disease; 5) atrial fibrillation from transient reversible cause (e.g., during respiratory tract infection or bronchospasm); 6) ongoing participation in a clinical trial; 7) life expectancy less than 3 years; 8) pregnancy; 9) inability to attend scheduled follow-up appointments; 10) refusal to join the study; and/or, 11) current hospitalization or hospitalization within 1 month prior to inclusion in the study.

Baseline demographic and clinical data were collected and recorded. Patients were followed-up at 6, 12, 18, 24, 30, and 36 months. Data relating to cardiovascular events, blood pressure, heart rate, and medications were collected at each follow-up visit. Data from each patient was written on a case record form and keyed into a web-based data collection and management system. The following data were collected: 1) demographic information; 2) history of stroke and bleeding; 3) type and duration of atrial fibrillation; 4) component parameters of CHADS2 score, CHA2DS2VASc score for stroke risk, and HAS-BLED score for risk of bleeding; 5) history of medical and cardiovascular disease; 6) antithrombotic medication; 7) reason for not using warfarin in those not taking warfarin; 8) concomitant medications; 9) twelve-lead ECG; and, 10) current INR. Protocols were established and followed by the data management team and statisticians to ensure the integrity and quality of the data before final analysis. Random site monitoring was also regularly performed. Approximately 70% of sites were audited. Data were collected during the 2014 to 2017 study period.

### Statistical analysis

Demographic and clinical data were interpreted using descriptive statistics. Continuous data are presented as mean ± standard deviation, and categorical data are shown as number and percentage. All statistical analyses were performed using SPSS Statistics version 20 (SPSS, Inc., Chicago, IL, USA).

## Results

A total of 3218 patients from 24 hospitals were included. Average age was 67.3 ± 11.3 years, and 1873 (58.2%) were male. Baseline demographic data, clinical characteristics, and use of antithrombotic medications are shown in Table 1. Average CHADS_2_, CHA_2_DS_2_-VASc, and HAS-BLED score was 1.8 ± 1.3, 3.0 ± 1.7, and 1.5 ± 1.0, respectively. One-hundred and three patients (3.2%) had history of radiofrequency ablation for atrial fibrillation. Among patients with coronary artery disease (CAD), 60 patients (1.9%) had history of percutaneous coronary intervention (PCI) within 12 months.

**Table 1:** Baseline characteristics of the study population and reasons for not using warfarin for those with CHA2DS2-VASc score ≥ 2

Variables	*N* = 3218
Age (years), mean ± SD	67.3 ± 11.3
Male gender, *n* (%)	1873 (58.2%)
Time after diagnosis of atrial fibrillation (years), mean ± SD	3.4 ± 4.4
Type of atrial fibrillation, *n* (%)	
- New	74 (2.3%)
- Paroxysmal	1001 (31.1%)
- Persistent	623 (19.4%)
- Permanent	1520 (47.2%)
History of heart failure, *n* (%)	875 (27.2%)
History of coronary artery disease, *n* (%)	505 (15.7%)
Devices, *n* (%)	330 (10.3%)
History of transient ischemic attack, *n* (%)	121 (3.8%)
History of ischemic stroke, *n* (%)	451 (14.0%)
Hypertension, *n* (%)	2183 (67.8%)
Diabetes mellitus, *n* (%)	777 (24.1%)
History of bleeding, *n* (%)	308 (9.6%)
Antithrombotic medications, *n* (%)	
Antiplatelet	854 (26.5%)
- Aspirin	761 (88.0%)
- ADP/P2Y12 inhibitors	191 (22.2%)
Anticoagulant	2422 (75.3%)
- Warfarin	2202 (90.9%)
- Direct thrombin inhibitor	80 (3.3%)
- Factor Xa inhibitors	140 (5.8%)
CHADS_2_ score, *n* (%)	
- 0	479 (14.9%)
- 1	955 (29.7%)
- 2	977 (30.4%)
- 3	480 (14.9%)
- 4	237 (7.4%)
- 5	79 (2.5%)
- 6	11 (0.3)
CHA2DS2-VASc score, *n* (%)	
- 0	207 (6.4%)
- 1	419 (13.0%)
- 2	674 (20.9%)
- 3	736 (22.9%)
- 4	589 (18.3%)
- 5	365 (11.3%)
- 6	163 (5.1%)
- 7	51 (1.6%)
- 8	13 (0.4%)
- 9	1 (0%)
HAS-BLED score, *n* (%)	
- 0	458 (14.2%)
- 1	1190 (37.0%)
- 2	1067 (33.2%)
- 3	403 (12.5%)
- 4	84 (2.6%)
- 5	15 (0.5%)
- 6	1 (0%)
Main reasons for not using warfarin, *n* (%)	653 (20.3%)
- Already taking anti-platelet drugs	174 (26.6%)
- Patient preference	151 (23.1%)
- Using NOACS	148 (22.7%)
- Bleeding risk	90 (13.8%)
- Physician preference	89 (13.6%)
- Fall risk	27 (4.1%)
- Warfarin compliance concern	22 (3.4%)
- Taking medication contra-indicated or cautioned for use with Warfarin	6 (0.9%)
- Allergy	1 (0.2%)

**Fig. 1:**
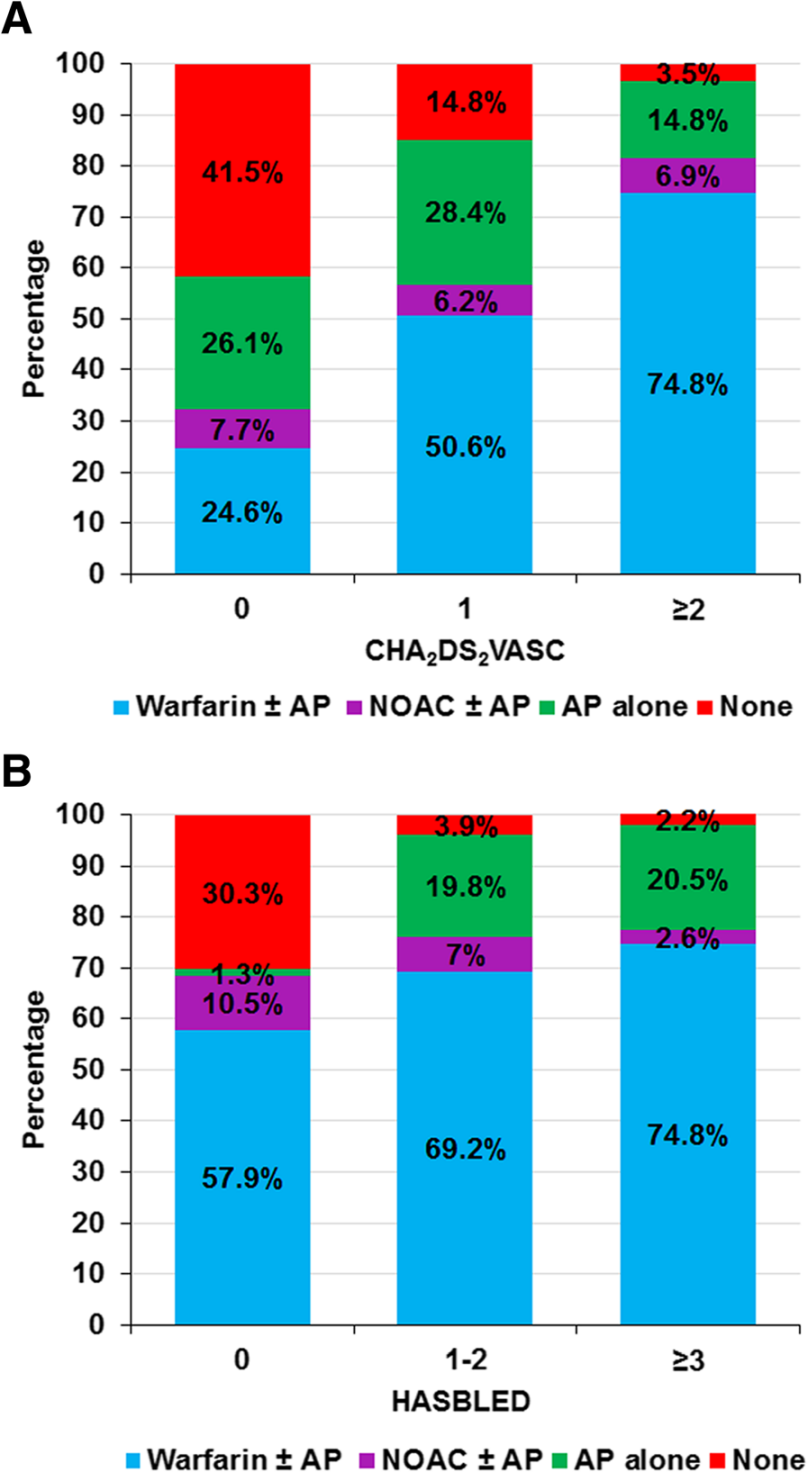
Use of antithrombotic treatment stratified by CHA_2_DS_2_-VASc score (**a**) and HAS-BLED (**b**) score. (Abbreviations: AP, antiplatelet; NOAC, non-vitamin K antagonist oral anticoagulant)

Antiplatelet and anticoagulant was used in 854 (26.5%) and 2422 (75.3%) patients, respectively. Anticoagulant alone was used in 2125 (66.0%) patients. Antiplatelet alone was prescribed in 557 (17.3%) patients, and used in combination with anticoagulant in 297 (9.2%) patients. Two hundred and thirty-nine (9.2%) patients were taking no antithrombotic medications. Figure 1 describes the rate of use of antithrombotic agents in patients with different CHA_2_DS_2_-VASc and HAS-BLED scores. The rate of anticoagulant use increased in patients with a higher CHA_2_DS_2_-VASc score. Anticoagulant was used in 67 (32.3%) patients with a CHA_2_DS_2_-VASc of 0, in 238 (56.8%) patients with a CHA_2_DS_2_-VASc score of 1, and in 2117 (81.6%) patients with a CHA_2_DS_2_-VASc score of ≥2 (Fig. 1a). Increased risk of bleeding, as reflected by a higher HAS-BLED score, did not influence a reduction in the use of anticoagulant (Fig. 1b). Among those who received anticoagulant, 2202 (90.9%) used warfarin and 220 (9.1%) used NOACs. When we analyzed the rate of NOAC use stratified by year of recruitment, an increase in the rate of NOAC use from 1.9% in 2014 to 25.6% in 2017 was observed (Additional file 1).

The reasons for not using warfarin in patients with a CHA_2_DS_2_-VASc score ≥ 2 that were not taking warfarin are shown in Table 1. The main reasons included already taking antiplatelet in 174 (26.6%) patients, patient preference not to take warfarin in 151 (23.1%), and current use of NOACs in 148 (22.7%).

## Discussion

In this study of 2014–2017 data from a multicenter registry in Thailand for patients with NVAF, we found a rate of anticoagulant use of 75.3%. However, only 41.8% of NVAF patients on warfarin had an INR within the 2–3 therapeutic range.

The baseline profiles of our study population are similar to the profiles described in previous reports [15–18]. The higher rate of anticoagulant use of 75.3% in this study compared to previous publications [13, 19] may be due to the implementation of clinical practice guidelines for the management of patients with NVAF [5, 20, 21].

GARFIELD AF enrolled patients with newly diagnosed NVAF starting with Cohort 1 in 2010–2011 [22, 23]. Only 56% of patients in Cohort 1 received anticoagulant. The rate of anticoagulant use markedly increased in 2015 [24]. Asian population in the GARFIELD registry had a lower percentage of anticoagulant use when compared to other regions of the world (38% vs. 53%) [25].

Data from the initial phase of GLORIA AF during 2011 to 2013 indicated that the rate of anticoagulant use was only 33% [26]. Anticoagulant use increased to 80% during phase 2, which enrolled patients until 2014. There was a difference in the rate of anticoagulant use (90% vs. 52%) and NOAC use (52% vs. 28%) between Western and Asian populations in GLORIA phase 2 [27]. Anticoagulant use in the present study was greater than the rate among Asian population in GLORIA phase 2, but the use of NOACs in our study was lower. In addition to China – Japan, Korea, Taiwan, and Singapore participated in GLORIA – all of which are high income Asian countries. In many other low to middle income countries like Thailand, governments limit the use of and reimbursement for new and expensive drugs. Regardless, our data shows an increase in the use of NOACs over time by year of enrollment.

Other registries in Western population include the ORBIT AF registry [16], which was conducted in the US during 2010 to 2011, and EORP AF, which was conducted in European countries [18]. Both studies confirmed a high rate of anticoagulant use (76% and 80%, respectively). However, among very low-risk patients (i.e., CHA_2_DS_2_-VASc 0), the rate of anticoagulant use from previous reports is 38–56% [16, 18, 22], which is close to consistent with the 32% rate of use found in the present study. Some patients may be on anticoagulation for reasons that include pre-cardioversion and/or post-cardioversion anticoagulation therapy, or they might have some degree of left ventricular systolic dysfunction, but they did not fit the criteria for CHA_2_DS_2_-VASc scor. This data also suggests that physicians fear stroke, even in patients at very low risk. In very low-risk patients, especially when young, an anatomical approach should be considered to delay relapse and to maintain sinus rhythm in order to reduce the need for OAC [28].

We herewith propose some possible explanations regarding why we observed a relatively high rate of anticoagulant use in this study. First, our registry is more recent. Second, almost all of the patients included in this study were managed by cardiologists, which may provide better care for patients with NVAF than non-cardiologists [29]. Third, most of the centers that participated in this registry are tertiary care hospitals.

Reasons for not using warfarin in this study included taking antiplatelet in 26.6%, patient preference (or patient prefers not to take anticoagulants) in 23.1%, and current use of NOACs in 22.7%. The main reason for not using warfarin from the GARFIELD registry [22] was physician’s choice (48.3%). Already taking antiplatelet is the reason for not using warfarin in only 7.2% of patients in the GARFIELD registry. This rate is much lower than the rate from our study, which indicates that the use of antiplatelet remains more common among Asian population.

This study has some mentionable limitations. First, our study population was enrolled mainly from university hospitals or large regional hospitals, which limits the generalizability. Second, we were unable to correlate the findings of this study with clinical outcomes since the 3-year follow-up data acquisition process is not yet completed.

## Conclusion

Antithrombotic drugs were prescribed in 75.3% of patients with NVAF. Among those who received anticoagulant, 90.9% used warfarin and 9.1% used NOACs.

## Additional file

Additional file 1: Rate of NOAC use stratified by year of recruitment. Rate of NOAC use increases as the year of enrollment more recent. (PDF 69 kb)

## Abbreviations

INR: International normalized ratio
IRB: Institutional review board
NOAC: Non-vitamin K antagonist oral anticoagulant
NVAF: Non-valvular atrial fibrillation
TIA: Transient ischemic attack
