# Supplementary material for: Risk profiles and pattern of antithrombotic use in patients with non-valvular atrial fibrillation in Thailand: a multicenter study
Source: BMC Cardiovasc Disord. 2018 Aug 25;18:174. doi: 10.1186/s12872-018-0911-4 (PMC6109333; doi:10.1186/s12872-018-0911-4)
Supplement: Supplementary file 1 — Rate of NOAC use stratified by year of recruitment. Rate of NOAC use increases as the year of enrollment more recent. (PDF 69 kb) [file 12872_2018_911_MOESM1_ESM.pdf]

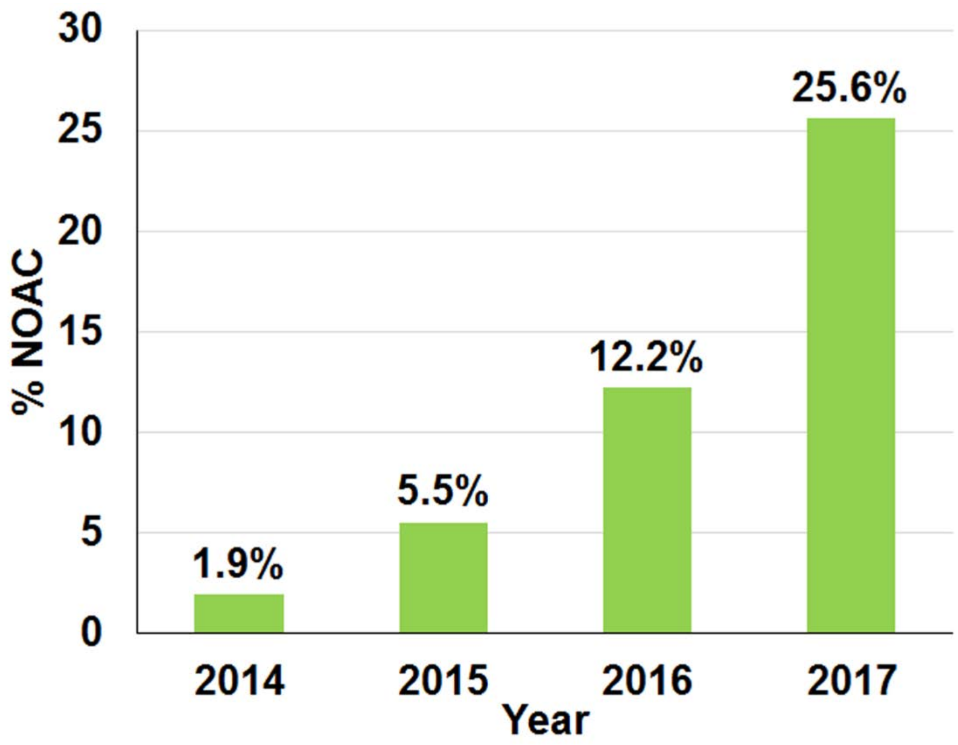

Additional File 1. Rate of NOAC use stratified by year of recruitment. Rate of NOAC use increases as the year of enrollment more recent.
